# Clinical Characteristics and Prognostic Factors of Posterior Segment Intraocular Foreign Body: Canadian Experience from a Tertiary University Hospital in Quebec

**DOI:** 10.1155/2021/9990290

**Published:** 2021-05-17

**Authors:** Jean-Philippe Rozon, Guillaume Lavertu, Mélanie Hébert, Eunice You, Serge Bourgault, Mathieu Caissie, Eric Tourville, Ali Dirani

**Affiliations:** ^1^Faculty of Medicine, Université Laval, Québec, Canada; ^2^Department of Ophthalmology, Centre Universitaire d'Ophtalmologie, CHU de Québec–Université Laval, Québec, Canada

## Abstract

**Purpose:**

To identify predictive factors for visual outcomes of patients presenting with a posterior segment intraocular foreign body (IOFB).

**Methods:**

A retrospective chart review was performed for all consecutive patients operated for posterior segment IOFB removal between January 2009 and December 2018. Data were collected for patient demographics, clinical characteristics at presentation, IOFB characteristics, surgical procedures, and postoperative outcomes. A multiple logistic regression model was built for poor final visual acuity (VA) as an outcome (defined as final VA 50 letters or worse [Snellen equivalent: 20/100]).

**Results:**

Fifty-four patients were included in our study. Ninety-three percent of patients were men, with a mean age of 40.4 ± 12.6 years. Metallic IOFB comprised 88% of cases with a mean ± standard deviation (SD) size of 5.31 ± 4.62 mm. VA improved in 70% of patients after IOFB removal. Predictive factors for poor VA outcome included poor baseline VA, larger IOFB size, high number of additional diagnoses, an anterior chamber extraction, a second intervention, the use of C3F8 or silicone tamponade, and the presence of vitreous hemorrhage, hyphema, and iris damage. Predictive factors for a better visual outcome included first intention intraocular lens (IOL) implantation and the use of air tamponade. In the multiple logistic regression model, both baseline VA (*p* = 0.009) and number of additional complications (*p* = 0.01) were independent risk factors for a poor final VA.

**Conclusions:**

A high number of concomitant complications and poor baseline VA following posterior segment IOFB were significant predictive factors of poor visual outcome.

## 1. Introduction

Despite growing prevention efforts, ocular injuries remain a major cause of blindness with an estimated incidence of 2.4 million cases in the United States [1]. Among these, intraocular foreign bodies (IOFB) continue being a major cause of visual decrease and blindness in the working population [2, 3], accounting for approximately 17% to 41% of penetrating ocular injury [4–7]. IOFB are most commonly of metallic type and generated from hammering, and a majority (59–88%) of IOFB affect the posterior segment [3, 8]. Despite newer techniques and advances in vitreoretinal surgery, many patients with posterior segment IOFBs still have poor visual outcomes, with a final visual acuity (VA) of less than 6/60 in 5% to 31% of patients [9, 10]. Significant challenges are associated with these interventions and the proper management of posterior segment IOFB remains a difficult issue.

The aim of this study is to identify predictive factors for visual outcomes of patients presenting with a posterior segment IOFB treated in a tertiary university hospital in Quebec, Canada, and to review the surrounding literature.

## 2. Methods

This retrospective study was approved by the Institutional Review Board of the Centre Hospitalier Universitaire de Quebec–Université Laval and adheres to the tenets of the Declaration of Helsinki.

All consecutive patients treated, hospitalized, or operated for posterior segment IOFB at the CHU de Quebec–Université Laval, a university tertiary hospital, between January 1, 2009, and December 31, 2018, were included in the study. Exclusion criteria included nonposterior segment IOFB and patients who had immediate exenteration, enucleation, or evisceration.

Data collection included age, gender, VA preoperatively and at least one month after the last intervention, final diagnosis, IOFB characteristics (localization, size, nature, and entry site), and delay between the injury and the surgery in days. The type and date of interventions, extraction type (pars plana or anterior chamber), surgical procedure (vitrectomy, buckle, endolaser photocoagulation, type of tamponade, use of a corneal graft, and phacoemulsification (none, first or second intention)), intraocular lens implantation (none, primary or secondary lens implantation), the number and the type of additional diagnoses and complications at presentation (e.g., endophthalmitis, cataract, hyphema, vitreous hemorrhage, retinal tear, retinal detachment, iris damage, choroidal detachment, or hemorrhage), the need for a second intervention (excluding phacoemulsification and intraocular lens implantation (phaco-IOL)), and an evisceration or enucleation were also collected. The context and mechanism of the injury as well as the presence of a relative afferent pupillary defect (RAPD) were not collected because of incomplete data related to these variables.

The surgical technique used to remove IOFB depended on clinical characteristics but usually involved a pars plana vitrectomy with IOFB extraction using forceps or magnets, followed by the use of a tamponade. Primary repair also varied depending on clinical characteristics but was generally performed using permanent suture of varied size depending on the localization of the entry wound.

### 2.1. Statistical Analysis

Data are presented as mean ± standard deviation (SD) for continuous variables and as frequencies (percentages) for categorical variables. Best corrected visual acuity (BCVA) was converted to an Early Treatment Diabetic Retinopathy Study (ETDRS) letter score from the metric Snellen scale for analysis. For the purposes of analyses, missing data for delay before presentation was imputed using the median value for the 5 (9%) missing cases. Patients were separated into two groups based on final VA: the good VA group with vision greater than 50 ETDRS letters (Snellen equivalent: 20/100) and the poor VA group with vision less than or equal to 50 ETDRS letters. Comparisons of both groups were performed to identify characteristics associated with a poor final VA. The following prognostic factors were analyzed for possible associations with final VA: age, time of injury to intervention, entry site, IOFB characteristics (i.e., location, size, nature), extraction site, clinical features on initial presentation (number of preoperative additional diagnoses, the presence of cataract, retinal tear, retinal detachment, vitreous hemorrhage, hyphema, iris damage, choroid detachment, endophthalmitis), IOL implantation (none, first or secondary implantation), the need for a secondary intervention (excluding phaco-IOL), and the need for a scleral buckle. Any damage to the iris that could reasonably be associated with the trauma was considered as an iris damage, regardless of size or severity. Characteristics and variables were compared between the two groups using independent Student's *t*-test or Mann-Whitney *U* test as appropriate for continuous variables and chi-square analysis for categorical variables. Shapiro-Wilk test and Q-Q plots with 95% confidence intervals were used to test for normal distribution of continuous variables.

A multiple logistic regression model was built to identify risk factors for worse final VA. A backwards elimination strategy was used to select variables with variables *p* > 0.2 removed. Given the small sample size, variables were included in the model while respecting a target of 5–9 events per predictor variable in building the models [11]. Age was forced into the model given its known influence on VA. Given the small proportion of each type of additional diagnoses found at baseline, these were combined in a single variable for the number of additional diagnoses. Indeed, we investigated whether a high number of additional diagnoses were associated with weaker visual prognosis. This factor was easily determined in clinic by the sum of all diagnosis or complications among these of which the patient was affected: cataract, vitreous hemorrhage, hyphema, retinal detachment, retinal tears, iris damage, choroid detachment, and endophthalmitis. Likewise, size of IOFB was not added to the model given the significant number of missing data (*n* = 22, 41%). Statistical analyses were performed using *R* for Windows, version 3.6.3 (R Foundation for Statistical Computing) and IBM SPSS Statistics for Windows, version 25.0 (IBM Corp., Armonk, NY). Statistical significance was set at *α* = 0.05.

## 3. Results

**Figure 1:**
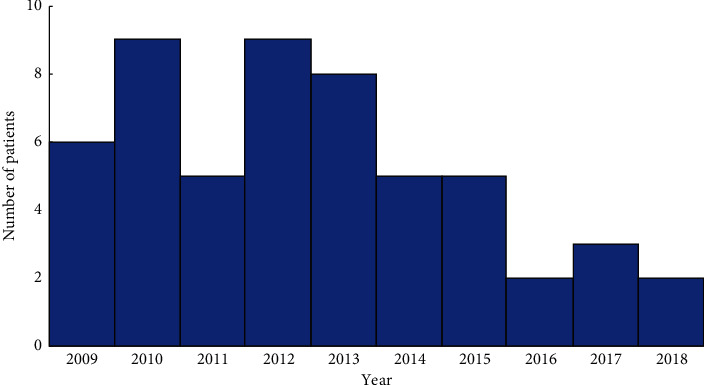
Histogram illustrating number of intraocular foreign bodies per year during the study period.

A total of 54 patients were included in the study (Table 1). There were 50 males (92.6%) and 4 females (7.4%), with a mean ± standard deviation (SD) age of 40.4 ± 12.6 years (range: 18–71). There was an average of 6.5 cases per year which decreased with time (Figure 1). Most IOFBs (*n* = 48, 89%) were metallic in nature. The exact nature of the metal involved, i.e., magnetic or not, was insufficiently documented. The size of the IOFB (data collected using pathology report preferably) ranged from 1 to 17 mm in its largest diameter, with a mean size of 5.31 ± 4.62 mm. The most common entry site was the cornea (*n* = 30, 55%), followed by the sclera (*n* = 15, 28%) and corneoscleral junction (*n* = 8, 15%). Most IOFB were intraretinal (*n* = 31, 57%) or located in the vitreous (*n* = 19, 35%). The delay between the injury and the intervention ranged from under 24 hours to 330 days with a median [first quartile, third quartile] of 1 [0, 2] days. Seventy-two percent of patients (*n* = 39/54) were operated ≤48 hours from injury.

The most common additional diagnoses and complications seen with IOFB were cataracts (*n* = 39, 72%), vitreous hemorrhages (*n* = 28, 52%), hyphemas (*n* = 17, 31%), retinal detachments (*n* = 16, 30%), retinal tears (*n* = 8, 15%), iris damage (*n* = 6, 11%), choroidal detachment (*n* = 3, 6%), and endophthalmitis (*n* = 1, 2%).

### 3.1. Visual Acuity before and after Surgery

Thirty-three percent of patients presented with an initial VA of 6/18 or better and 15% of patients had an initial VA of 6/9 or better. Forty-four percent of patients presented with an initial VA of no light perception (NLP), light perception (LP), or hand motion (HM). On final presentation, 50% of patients had a VA of 6/18 or better and 33% of patients had a final VA of 6/9 or better. Moreover, only 19% of patients had a final VA of NLP, LP, or HM (Table 2). Accordingly, 70% of cases had an improvement of VA after the removal of IOFB. The difference between the initial and the final VA was statistically significant (*p* = 0.013). In four cases (7%), the VA remained stable, while in 12 cases (22%), the VA worsened.

### 3.2. Surgery

Among the 54 patients, 21 (39%) received surgical intervention within 24 hours of the incident. IOFB removal was achieved via the pars plana in 48 cases (89%) or via the anterior chamber in 5 cases (9%). All patients underwent a vitrectomy. Nineteen patients (35%) had a scleral buckle (combined to the vitrectomy) and 5 (9%) had a corneal graft. 43 patients (80%) underwent phacoemulsification. Thirty-two eyes (59%) had phacoemulsification with secondary intraocular lens (IOL) implantation while only 20% (*n* = 11) had a first intention IOL implantation. 14 patients (26%) underwent a second intervention (excluding phacoemulsification with intraocular lens (IOL) implantation). Second interventions performed mainly included vitrectomy (with or without combined buckle) used in case of complications following the primary intervention, such as retinal detachments with or without vitreoretinal proliferation. The intraocular tamponade used during the surgery was air (43%), SF6 (28%), C3F8 (13%), and silicone oil (17%).

### 3.3. Predictive Factors for the Final Visual Outcomes

The results revealed that poor final BCVA was associated with the following factors: a poor baseline VA (*p* < 0.001), a larger IOFB size (*p* < 0.001), a higher number of additional diagnoses (*p* < 0.001), the presence of vitreous hemorrhage (*p* = 0.002), hyphema (*p* < 0.001), iris damage (*p* = 0.042), an anterior chamber extraction (*p* = 0.009), a second intervention (*p* = 0.018), and the use of C3F8 (*p* = 0.002) or silicone tamponade (*p* = 0.027) (Table 3). Likewise, a good final VA was significantly associated with a first intention IOL implantation (*p* = 0.026) and air tamponade (*p* = 0.019).

For further clarification of independent risk factors for a poor visual outcome, a multiple logistic regression model was built (Table 4). Independent risk factors of poor final VA (i.e., less than or equal to 50 ETDRS letters) were worse baseline VA (*p* = 0.009) and a higher number of additional diagnoses/complications at presentation (*p* = 0.01).

## 4. Discussion

Despite growing awareness for the use of protective eyewear, IOFBs continue to be a common cause of blindness in both developed and undeveloped countries [1]. We describe a case series of 54 patients undergoing IOFB extraction over 10 years. There was a gradual decrease in annual IOFB numbers, but these remain a significant cause of severe vision loss.

As in previous studies, patients are predominantly male with an average age of 40 years [1, 12, 13]. In one study, 50–54% of IOFBs were reported as work-related, with home projects accounting for only a minority (13%) of injuries [8]. Only 0.77 to 6% of patients diagnosed with an IOFB reported wearing protective eyewear during the incident [8, 14–16]. Thus, safety and preventive measures at work should continue to be enforced.

### 4.1. Intraocular Foreign Body Characteristics

The majority (88%) of IOFBs in our study were inorganic and metallic in origin. In general, inorganic, metal IOFBs are better tolerated than organic matter. Most metals are inert and, if small and deeply lodged, may be left in place [17]. Hammering also tends to be the main mechanism in 71–80% of cases [8], which typically generates relatively small foreign bodies and minimal ocular trauma [16]. Meanwhile, organic IOFBs such as wood, vegetation, or animal matter have a high risk of infection and inflammation [1]. Only one patient (2%) in this study had an organic IOFB, which may partially explain the low incidence of endophthalmitis (*n* = 1, 2%) though this patient did not develop endophthalmitis.

We also found a significant difference in IOFB size based on final visual prognosis (final VA > 50 letters = 2.8 ± 1.7 mm vs. final VA ≤50 letters = 7.5 ± 5.2 mm, *p* < 0.001). Smaller IOFBs (defined as <5 mm by Liu et al. [1]) have also been correlated with a better visual outcome in several studies [1, 9, 10, 18]. Larger IOFBs have been associated with more complications, including cataracts, hemorrhage, and uveal prolapse, resulting in a worse final VA [12].

There was no significant association between entry site and visual prognosis. In contrast, an earlier study reported better visual prognosis with a scleral or corneoscleral entry site than a corneal site [19].

### 4.2. Complications and Visual Outcome

Complications identified as poor prognostic factors in our study were consistent with findings in the literature, including vitreous hemorrhage [10, 16] and hyphema [10]. Both presence of vitreous hemorrhage and hyphema are typically indicative of greater traumatic force to the ocular structures. On the long-term, this also translates into poor visual outcomes through proliferative vitreoretinopathy (PVR) for which vitreous hemorrhage is the strongest stimulus [20]. We found a significant association with iris traumas, which was not identified in previous studies due to low incidence limiting statistical power [1, 9, 13, 14]. Retinal detachment at presentation is also associated with poor visual outcome with development of PVR [8–10, 19, 21]. In this study, this did not reach statistical significance (*p* = 0.08), but the extent of retinal detachment could differ between outcome groups. On the contrary, cataract development does not seem to influence final visual outcomes in our study and in others [12, 18], given the availability of phacoemulsification with intraocular lens implantation. Other identified risk factors include endophthalmitis [8, 12, 22], RAPD [10, 16], retinal breaks [12, 13], PVR [10, 22], and prolapse of intraocular tissue [8, 10]. We were underpowered to detect any effect of endophthalmitis and retinal tears, in which there were respectively 1 and 8 cases. Lastly, a greater number of complications were highly associated with a poor visual prognosis in this study given that this reflects greater damage to ocular structures.

### 4.3. Timing of Intervention

There are many conflicting studies regarding the management of IOFB in terms of the optimal time of removal. Recent studies suggest that immediate IOFB removal may not be as critical to vision preservation as previously thought [12, 23]. A large retrospective study conducted in China reported a longer time (average 31.9 days) between injury and removal compared to most previous studies without negatively impacting the visual outcome [12]. In addition, due to the difficulty associated with complete removal of the posterior hyaloid and the increased risk of intraoperative bleeding in an inflamed eye, delaying surgical intervention may be preferable in young patients [12].

Our study found no association between the time before removal and the final visual outcome. These results are consistent with Wickham et al. [10], Liu et al. [12], and Nicoara et al. [21]. However, given that the majority of patients (72%) were operated ≤48 hours from injury, there may not be enough patients with longer delays to determine its effect. In contrast, Chaudhry et al. reported that early intervention reduced the risk of endophthalmitis, which could impact the visual prognosis [24]. Although it was not possible to evaluate this association in our study due to our low incidence of endophthalmitis. It is possible that the short delay between injury and intervention in most of our patients may be contributory. In our center, we continue to prioritize these patients, and generally vitrectomy and IOFB removal are carried out within the first 24 hours of presentation.

### 4.4. Prognostic Factors for Visual Outcome

Only one-third of the patients in our study presented with an initial VA of 6/18 or better and 15% of patients had an initial VA of 6/9 or better. We found that better initial VA was associated with better final VA. This correlation has previously been found in numerous studies [10, 12, 13].

The requirement for repeated surgery is associated with a poorer visual outcome [13, 25, 26]. We also identified several factors of poor visual prognosis which were not as evaluated in the literature given small sample sizes in other studies. The use of a secondary IOL implantation and the need for a second intervention (excluding phacoemulsification and IOL implantation) were both found to be significant predictive factors for worse visual outcomes. Delayed IOL implantation often occurs in cases associated with greater ocular trauma and/or impairment of zonular support. Moreover, additional surgery implies more severe ocular trauma [12]. An anterior chamber extraction as compared to pars plana was likewise found to be a prognostic factor of poor visual outcome. It is probably due to the larger sizes of the IOFBs or to the greater structural breakdown when the FB passes through the anterior chamber in the case of an anterior chamber extraction.

The use of C3F8 gas or silicone oil tamponades compared to air tamponade was associated with poor visual outcome in this study. Similar results were reported by Liu et al. with C3F8 [12] and Akesbi et al. with gas tamponade compared to silicone oil or no tamponade [19]. However, it is important to note that, in this study and in other studies, C3F8 and silicone oil were often used in more serious traumas and in cases associated with retinal reattachment, and this association is therefore subject to indication bias [18].

### 4.5. Limitations

This is a retrospective study with a relatively small sample size that provides epidemiological data for IOFB in our region. Our results are comparable to previous studies in other regions, including a study in North Carolina in which similar factors of poor visual outcomes (i.e., poor initial VA, the presence of a RAPD, and vitreous hemorrhage) were identified among a cohort of 59 predominantly young male patients [16]. Strengths of this study include the multiple logistic regression which allowed us to identify independent risk factors for poor final VA. The detailed description of additional diagnoses and complications at presentation allows for a better appreciation of the severity of the cases included in our study as well as providing a large analysis of predictive factors of visual outcome.

In conclusion, this study identified multiple risk factors for poor VA following posterior segment IOFB over the span of a decade. Independent risk factors included worse baseline VA and a high number of concomitant complications (e.g., vitreous hemorrhage, hyphema, iris damage, and cataract). These factors will allow ophthalmologists to more accurately assess the trauma severity and visual prognosis of patients with a posterior segment IOFB. Future directions include assessing the impact on long-term visual acuity and the impact of the type of intervention on long-term visual prognosis.

## Figures and Tables

**Table 1:** Clinical characteristics of 54 study participants.

Clinical characteristics	Mean ± SD or *n* (%)
IOFB size, mm	5.31 ± 4.62
IOFB nature	
Metallic	48 (89%)
Glass	3 (6%)
Fireworks	1 (2%)
Organic	1 (2%)
Unknown	1 (2%)

IOFB entry site	
Cornea	30 (56%)
Sclera	15 (28%)
Corneoscleral	8 (15%)
Unknown	1 (2%)

IOFB location	
Retina	31 (57%)
Vitreous	19 (35%)
Orbit	2 (4%)
Optic nerve	1 (2%)
Unknown	1 (2%)

Secondary clinical features	
Cataract	39 (72%)
Vitreous hemorrhage	28 (52%)
Hyphema	17 (31%)
Retinal detachment	16 (30%)
Retinal tears	8 (15%)
Iris damage	6 (11%)
Choroid detachment	3 (6%)
Endophthalmitis	1 (2%)

IOFB, intraocular foreign body; SD, standard deviation.

**Table 2:** Initial and final VA.

VA	Initial VA *n* (%)	Final VA *n* (%)
NLP, LP or HM	24 (44%)	10 (19%)
CF to 6/60	8 (15%)	16 (30%)
6/45 to 6/21	4 (7%)	1 (2%)
6/18 to 6/12	10 (19%)	9 (17%)
6/9 to 6/6	8 (15%)	18 (33%)

CF, counting fingers; HM, hand motion; LP, light perception; NLP, no light perception; VA, visual acuity.

**Table 3:** Baseline characteristics of 54 patients with intraocular foreign body by final visual acuity.

Characteristics	Final visual outcome		*p* value
	Good >50 letters ETDRS	Poor ≤50 letters ETDRS	
	Mean ± SD or *n* (%)		
Male sex	28 (93%)	22 (92%)	0.82
Age	38.5 ± 12.5	43.5 ± 12.1	0.15
Baseline VA	57.9 ± 30.9	12.5 ± 24.3	**<0.001**
Delay	20.0 ± 66.7	9.8 ± 39.3	0.47
IOFB			
Metallic nature	28 (93%)	19 (83%)	0.22
Size	2.8 ± 1.7	7.5 ± 5.2	**<0.001**
Localization			
Vitreous	13 (43%)	6 (25%)	0.16
Retina	17 (57%)	14 (58%)	0.90
Entry site			
Cornea	17 (57%)	13 (57%)	0.99
Corneoscleral	3 (10%)	5 (22%)	0.24
Sclera	10 (33%)	5 (22%)	0.35
Number of additional diagnoses	1.7 ± 1.0	3.5 ± 1.4	**<0.001**
Vitreous hemorrhage	10 (33%)	18 (75%)	**0.002**
Cataract	20 (67%)	19 (79%)	0.31
Retinal detachment	6 (20%)	10 (42%)	0.08
Retinal tear	3 (10%)	5 (21%)	0.27
Hyphema	3 (10%)	14 (58%)	<**0.001**
Iris damage	1 (3%)	5 (21%)	**0.042**
Choroid detachment	1 (3%)	2 (8%)	0.43
Endophthalmitis	0 (0%)	1 (4%)	0.26
Interventions			
Anterior chamber extraction	0 (0%)	5 (21%)	**0.009**
Pars plana extraction	30 (100%)	24 (100%)	1.00
Scleral buckle	8 (27%)	11 (46%)	0.14
Graft	2 (7%)	3 (13%)	0.46
First intention IOL	8 (27%)	3 (13%)	**0.026**
Reoperation	4 (13%)	10 (42%)	**0.018**
Tamponade			
Air	17 (57%)	6 (25%)	**0.019**
SF6	11 (37%)	4 (17%)	0.10
C3F8	0 (0%)	7 (29%)	**0.002**
Silicone	2 (7%)	7 (29%)	**0.027**
Postoperative VA	79.6 ± 7.0	13.1 ± 14.9	<**0.001**

Bolded figures = statistically significant results at the 0.05 level. Abbreviations: ETDRS, Early Treatment Diabetic Retinopathy Study; IOFB, intraocular foreign body; SD, standard deviation; VA, visual acuity.

**Table 4:** Multiple logistic regression for predictive factors of worse final visual acuity following intraocular foreign body extraction in 54 patients.

Characteristics	OR (95% CI); *p* value
Age, years	1.00 (0.93, 1.07); 0.97
Baseline VA, ETDRS	0.97 (0.94, 0.99); **0.009**
Number of additional diagnoses	2.95 (1.40, 7.56); **0.01**

Bolded figures = statistically significant results at the 0.05 level. Abbreviations: CI, confidence interval; ETDRS, Early Treatment Diabetic Retinopathy Study; OR, odds ratio; VA, visual acuity.

## Data Availability

All the data relevant to the study are included in the article. The datasets used and/or analysed during the present study are available from the corresponding author on reasonable request.
